# Supplementary material for: A register-based study of long-term health and social care costs among children with prenatal alcohol exposure
Source: PLoS One. 2026 Mar 11;21(3):e0332113. doi: 10.1371/journal.pone.0332113 (PMC12978448; doi:10.1371/journal.pone.0332113)
Supplement: S1 Appendix — Health care unit costs, most common diagnoses. (PDF) [file pone.0332113.s001.pdf]

S1. Hospital care

S1.1 DRG price list

DRG prices were extracted for the 80% most common diagnoses and selected expensive rare diagnoses

Using Health care unit pricing list in Finland for years:

2017: https://www.julkari.fi/handle/10024/142882

2011: https://www.julkari.fi/handle/10024/114683

2006: https://www.julkari.fi/bitstream/handle/10024/76754/T3-2008-VERKKO.pdf

2001: https://www.julkari.fi/bitstream/handle/10024/76753/Aiheita1-2003.pdf?sequence=1&isAllowed=y

Prices for other years were extrapolated using the health care price index.

For diagnoses without DRG price:

- inpatient care was priced according to length of hospital stay multiplied by the average price for a hospital day for each specialty, and

- outpatient care was priced according to specialty clinic price.

In case of missing outpatient diagnosis, a regular pediatric clinic visit price was used for patients under the age of 16 years and a regular medicine visit price was used for patients aged 16 years and over, respectively.

S1 Table 1.1 List of used DRG prices in euros, nominal prices in each year before index correction

Diagnosis	2001 price	2006 price	2011 price	2017 price
Outpatient visit pediatric	178.8	223.5	257.2	279.1
Outpatient visit medicine	197.9	200.3	292.6	343.3
Outpatient visit pediatric neurology	249.1	161.4	289.2	303.7
Outpatient visit neurology	213.8	190.4	305.6	327.6
Outpatient visit pediatric psychiatry (mean price for regular visit and acute visit)	165.0	253.8	281.5	319
Outpatient visit adolescent psychiatry (mean price for regular visit and acute visit)	165.0	188.2	240	259.5
Outpatient visit adult psychiatry	143.2	157.5	162	173
Outpatient visit ear-nose-throat (ENT)	101.3	152.8	230.2	258.5
Outpatient visit ophthalmology	88.7	172.6	201.2	275
Outpatient visit skin and venereal diseases	99.7	211.7	199	229.4

Outpatient visit oncology	160.3	196.1	288.3	367.4
Outpatient visit surgery	191.6	169.9	266.7	325.7
Outpatient visit neurosurgery	191.6	158.3	316.8	363.2
Outpatient visit gynecology	148.6	192.8	248.6	283.3
Inpatient day pediatric	654.1	899.3	773.6	936.4
Inpatient day medicine	379.8	575.1	587.5	568.3
Inpatient day children psychiatry,	458.8	497.4	638	855
Inpatient day adolescent psychiatry	458.8	451.1	568	685
Inpatient day adult psychiatry	247.7	265.9	408	437
Inpatient day pediatric neurology	554.0	919.0	708.7	768.2
Inpatient day neurology	345.7	484.2	535.8	390.7
Inpatient day gynecology and labor	461.3	697.2	577.9	746.5
Inpatient day surgery S00-S05, S07-T08, T10-T35	563.5	890.5	948.8	1107.7
Inpatient day neurosurgery	NA	1208.7	1507.2	1561.0
Inpatient day ENT	515.2	916.1	947.9	1104.7
Inpatient day ophthalmology	675.2	1040.3	873.3	1006.3
Inpatient day oncology	419.0	620.4	565.7	616.7
Inpatient day skin and venereal diseases	226.7	475.8	349.3	438.7
Inpatient day NICU intensive care	NA	2596.3	2568	3026
Inpatient care NICU surveillance care	NA	982.6	612	766
Prematurity extreme <1000g, P07.0 or P07.2	23824.5	137865.8	81009.1	136342.4
Prematurity birth weight 1000-1499g,	NA	32338.5	42276.4	50799.4
Prematurity other P07.3	4519.3	8520.6	11635.2	16253.0
Term infant severe complications	3236.5	12341.5	10971.9	14293.3
Term infant other complications, P00-P05, P08-P96	2583.9	2936	3813.7	5692.9
Gastroenteritis A08, A09	963.6	1395.8	1336.9	2455.8
Sepsis <16 years A41	1793.1	2109.2	3183.3	4945.9
Sepsis 16+ years A41	4812.1	5258.4	6207.4	5161.8
Upper respiratory or ear infection J00, J01, J06, H65-H66	849.8	1037.7	1197.2	3447
Eye disease or disorder H15-H59, Q10-Q16	829.5	1147.9	2215.7	3203.2
Congenital heart defect Q20-Q29	1246.2	3874.5	4396.6	5732.1
Pneumonia J11-J18	1524.7	2312.1	1834.3	2350.2
Laryngitis, tracheitis or tonsillitis J02-J05	NA	994.1	2753.5	4906.0

Hypertrophy of tonsils or adenoids or peritonsillar abscess J35-J36	862.0	905.1	1883,1	3189.3
Obstructive respiratory disease, J45.J46, J21	1315.3	1560.5	1654.2	2535.5
Pyelonephritis N10	1608.1	2173.4	1904.8	2370.9
Hernia K40-K46	935.2	1241.3	3557.0	9946.6
Appendicitis K35-K37	2169.2	2220.5	2897.6	4089.1
Seizure or headache R56, G40, R51,8, G43, G44	1642.6	2125.5	2514.3	4679.6
Hydrocephalus G91	5385.4	10871.7	10865.4	8652.6
Leukemia or lymphoma C81-C96, (first hospitalization, later as oncological days)	NA	11651.0	10518.7	10582.7
Brain tumor operation, first hospitalization, later as neurosurgical days), C71	5385.4	7462.5	10725.7	10151.3
Complicated central nervous system disorder, cerebral palsy, G80	2718.1	3705.2	4187.3	4063.2
Intellectual disability F70/F79	3419.5	2451.7	3075.5	4841.4
Neuropsychiatric disorders F84, F90-F95	3419.5	3461.7	3922.9	5435.9
Neurodevelopmental problems in childhood Q86, F80-F83	3419.5	3202.7	3003.5	6577.1
Depressive mood disorders F32-F39	876.2	1450.4	NA	4681.3
Anxiety F41	876.2	1793.1	NA	4215.8
Adjustment disorders F43	876.2	1136.8	1336	3056.6
Psychosis F20-F29	6389.7	4204.7	NA	4330.4
Eating disorder F50	NA	6637.3	5392.1	6563.5
Spontaneous abortion O03	904.5	768.3	776.3	1083.0
Medical abortion O04-O07	965.7	758.6	990.8	1738.9
Complications of labor and delivery O60-O75	1904.9	2609.3	2411.5	3030.2
Child delivery O80 uncomplicated	975.5	1663.5	1881.3	2270.9
Cesarean section delivery O82	2116.3	2366.8	3081.6	3584.2
Diabetes E10	1925.2	2741.2	2938.0	3620.7
Skin disorder <16 years L20	772.5	649.3	1516.8	2357.5
Allergic reaction L27, L50, T78	412.7	768.9	909.2	3735.2
Fracture of skull or intracranial injury except concussion S02, S06.1-S06.9	1171.0	2056.3	2995.6	2497.2
Concussion S06.0	512.3	717.4	916.3	2163.0

Cleft lip and palate Q35-Q37	2555.5	3510.0	5662.9	6475.5
Wrist, hand or foot injury S50-S69, S90-S99 <16 years	1526.8	1115.3	1272.1	2534.2
Wrist, hand or foot injury S60-S69, S90-S99 16+ years	788.8	990.9	758.7	1763.5
Upper arm or lower leg injury S40-S49 or S80-S89 <16 years	1313.3	1302.4	1334.4	2401.8
Upper arm or lower leg injury S40-S49 or S80-S89 16+ years	868.1	842.7	1244.6	1904-8
Poisoning or intoxication T36, T51 <16 years	546.9	868.0	1308.1	4448.1
Poisoning or intoxication T36, T51 16+ years	738.0	1350.9	1565	4547.0
Scoliosis operation M41 (only priced once/person) M41	NA	24350.6	14695.0	14667.0
Viral infection B00-B09, B25-B34 <16 years	1132.4	1763.6	1478.5	2619.8
Viral infection B00-B09, B25-B34 16+ years	2150.9	3483.1	3155.3	2522.5
Juvenile arthritis M08	1667.0	2492.1	3296.3	3364.4
Testis torsion N44	1433.3	1469.0	2296.8	3390.8
Congenital atresia or stenosis of GI-tract Q39-Q43	5659.8	6768.3	8552.5	8333.1

As the majority of hospitalizations occurred in children under the age of 16, mainly DRG prices for children are selected, when there are different prices for child and adult. For children there is typically just one DRG price per diagnosis group, when for adult there are prices for both uncomplicated and complicated diagnosis. For this reason, the DRG price for children is more straightforward. However, in case of significant difference in DRG price for adult and child, different prices for hospitalizations before and after the age of 16 were listed.

Estimations are conservative as regular prices and prices for uncomplicated visits were selected. Inpatient day costs are calculated by dividing the mean cost for inpatient care with the mean length of inpatient care.

For neonates with birthweight <1500g or neonatal diagnoses with DRG prices available, DRG prices were used.

Due to the lack of information concerning the length of neonatal hospital care, length of NICU stays for those with birth weight 1500g and over had to be estimated using the day when the mother was discharged from hospital as the day of discharge also for the infant. This unfortunately causes significant underestimation.

S1.2. List of diagnose groups for each specialty for outpatient visits and when specific DRG price for a diagnosis has not been selected

S1 Table 1.2 Diagnose groups without specific DRG price

Pediatric diagnoses	All hospitalizations for those under the age 16 years, which are not included in DRG list or other specialty groups below
Medicine diagnoses	All hospitalizations for those aged 16 years or more, which are not included in DRG list or other specialty groups below
Child psychiatry diagnoses	For those under 13 years F10-F69, F84-F98
Adolescent psychiatry diagnoses	For those between 13-17 years: F10-F69, F84-F95,
Adult psychiatry diagnoses	For those 18 years or more: F10-F69, F84-F95
Pediatric neurology diagnoses	For those under 16 years: F00-F09, F70-F83, G00-G90, G92, G98-G99, Q00-Q01, Q04, Q06, Q86-Q99
Neurology diagnoses	For those 16 years or more: F00-F09, F70-F83, G00-G90, G92, G98-G99, Q00-Q01, Q04, Q06, Q86-Q99
Gynecology and labor diagnoses	O00-O99
Oncology and hematology diagnoses	C00-C43, C45-C68, C73-C97, D0, D60-D77
Surgery diagnoses	S00-S99, T00-T35, N43-N45, N47, Q35-Q79, R10
Neurosurgery diagnoses	G91, G93-G97, D33, D43, C70-C72, Q03, Q05, Q07
ENT diagnoses	H60-H95, J35-J39, Q17, Q30-Q32
Ophthalmology diagnoses	C69, H00-H59, Q10-Q16
Skin and venereal diagnoses	A50-A69, L00-L99, R23, D22, D23
NICU intensive care	Intensive NICU in birth register, P07.0-P07.2, P20-P37, P50-P56, P60, P90, P91, P96.0
NICU surveillance care	Surveillance NICU in birth register, other P diagnoses

S1.3 The 50 most common diagnoses

S1 Table 1.3a The 50 most common diagnoses, in descending order of prevalence, in hospital care that differentiate PAE with FASD significantly from controls with RR ≥ 1.5, lower bound of 95% C.I. ≥1 and p-value < 0.05.

Diagnosis	PAE with FASD<br><br>N=48<br>n (%)	CONTROL<br><br>N=1795<br>n (%)	RR	95% CI.	P-value
H65/H66 Middle ear infections	33 (68.8)	456 (25.4)	2.7	2.2-3.3	< 0.0001
Q75 Malformations of skull	24 (50.0)	7 (0.4)	128.2	58.1-283.0	< 0.0001
Q18 Facial malformations	16 (33.3)	10 (0.6)	59.8	28.7-124.9	< 0.0001
Q38 Malformations of mouth	12 (25.0)	33 (1.8)	13.6	7.5–24.7	< 0.0001
A08 VIral gastroenteritis	12 (25.0)	146 (8.1)	3.1	1.8–5.1	< 0.0001
H50 Strabismus	11 (22.9)	32 (1.8)	12.9	6.9–23.9	< 0.0001
J21 Acute bronchiolitis	11 (22.9)	139 (7.7)	2.0	1.2–3.4	0.0118
J35 Chronic diseases of tonsils and adenoids	11 (22.9)	216 (12)	1.9	1.1-3.2	0.0180
F90 Disorders of attention and activity	10 (20.8)	76 (4.2)	4.9	2.7–8.9	<0.0001
F83 Mixed developmental disorders	10 (20.8)	48 (2.7)	7.8	4.1–14.5	<0.0001
Q10 Ptosis	9 (18.8)	13 (0.7)	25.9	11.6–57.6	<0.0001
P07 Prematurity	9 (18.8)	112 (6.2)	3.0	1.6–5.6	0.0005
P05 Small for gestational age	9 (18.8)	31 (1.7)	10.9	5.5–21.5	<0.0001
Q30 Nasal malformations	8 (16.7)	2 (0.1)	149.6	32.6–685.8	<0.0001
H52 Disorders of refraction and accommodation	8 (16.7)	27 (1.5)	11.	5.3–23.1	<0.0001
F98 Behavioral and emotional disorders of childhood	8 (16.7)	66 (3.7)	4.5	2.3–8.9	0.0005
F32 Depressive episodes	8 (16.7)	129 (7.0)	2.3	1.2–4.5	0.0117
Q21 Septal heart defects	7 (14.6)	31 (1.7)	8.4	3.9–18.2	<0.0001
F92 Mixed disorders of conduct and emotions	7 (14.6)	64 (3.6)	4.1	2.0–8.5	0.0001
F81 Learning disorders	7 (14.6)	79 (4.4)	3.3	1.6–6.8	0.0011
Q11 Microphthalmos	6 (12.5)	1 (0.1)	224.4	27.5–1828	<0.0001
F94 Disorders of social functioning in childhood	6 (12.5)	13 (0.7)	17.3	6.8–43.5	<0.0001
E34 Other endocrine disorders (short stature)	6 (12.5)	17 (0.9)	13.2	5.4–32.0	<0.0001
F93 Emotional disorders of childhood	6 (12.5)	74 (4.1)	3.0	1.4–6.6	0.0054
F70 Mild intellectual disability	5 (10.4)	10 (0.6)	18.7	6.6–52.6	<0.0001
R29 Other nervous and musculoskeletal symptoms	5 (10.4)	24 (1.3)	7.8	3.1-19.5	0.0007
H10 Conjunctivitis	5 (10.4)	34 (1.9)	5.5	2.2–13.4	0.0002
F82 Motor coordination disorder	5 (10.4)	52 (2.9)	3.6	1.5–8.6	0.0040
R62 Delayed development	5 (10.4)	52 (2.9)	3.6	1.5–8.6	0.0040
B34 Viral infection of unspecified site	5 (10.4)	51 (2.8)	3.7	1.5-8.8	0.0035
Q02 Microcephaly	4 (8.3)	- (0.0)	NA		<0.0001

Q14 Malformation of posterior segment of eye	4 (8.3)	1 (0.1)	149.6	17.0-1313	<0.0001
Q25 Malformation of great arteries	4 (8.3)	17 (0.9)	8.8	3.1-25.2	<0.0001
K07 Dentofacial anomalies	4 (8.3)	17 (0.9)	8.8	3.1-25.2	<0.0001
Q74 Other limb malformation	4 (8.3)	8 (0.4)	18.7	5.8-60.0	<0.0001
Q82 Other malformation of skin	4 (8.3)	24 (1.3)	6.2	2.2-17.3	0.0004
H90 Conductive and sensorineural hearing loss	4 (8.3)	34 (1.9)	4.4	1.6-11.9	0.0035
K40 Inguinal hernia	4 (8.3)	29 (1.6)	5.2	1.9-14.1	0.0014
PAE= Prenatal Alcohol Exposure, FASD= Fetal Alcohol Spectrum Disorders, RR= Risk Ratio, CI. Confidence Interval					

S1 Table 1.3b The 50 most common diagnoses, in descending order of prevalence, in hospital care that differentiate PAE without FASD significantly from controls with RR ≥ 1.5, lower bound of 95% C.I. ≥1 and p-value <0.05.

Diagnosis	PAE without FASD<br><br>n=379<br>n (%)	CONTROL<br><br>n=1795<br>n (%)	RR	95% C.I.	P-value
H65/H66 Middle ear infections	151 (39.8)	456 (25.4)	1.6	1.4–1.8	< 0.0001
F93 Emotional disorders of childhood	63 (16.7)	74 (4.1)	4.1	3.0–5.6	< 0.0001
F32 Depressive episodes	62 (16.4)	129 (7.2)	2.3	1.7–3.0	< 0.0001
J06 Acute upper respiratory infections	59 (15.6)	182 (10.1)	1.5	1.2–2.0	0.0020
F92 Mixed disorders of conduct and emotions	58 (15.3)	64 (3.6)	4.3	3.1–6.0	< 0.0001
F41 Anxiety disorders	50 (13.2)	103 (5.7)	2.3	1.7–3.2	< 0.0001
F43 Stress reactions and adjustment disorders	43 (11.3)	69 (3.8)	3.0	2.1–4.2	< 0.0001
F90 Disorders of attention and activity	42 (11.1)	76 (4.2)	2.6	1.8–3.8	< 0.0001
F91 Conduct disorders	33 (8.7)	33 (1.8)	4.7	3.0–7.6	< 0.0001
F98 Behavioral and emotional disorders of childhood	27 (7.1)	66 (3.7)	1.9	1.3–3.0	0.0028
P04 Prenatal exposure to noxious influences (except P04.3 = PAE)	25 (6.6)	4 (0.2)	29.8	10.4–85.0	< 0.0001
P05 Small for gestational age	25 (6.6)	31 (1.7)	3.8	2.3–6.4	< 0.0001
F94 Disorders of social functioning in childhood	24 (6.3)	13 (0.7)	8.7	4.5–17.0	< 0.0001
F10 Alcohol use disorders	23 (6.1)	24 (1.3)	4.5	2.6–8.0	< 0.0001
R62 Developmental delay	22 (5.8)	52 (2.9)	2.0	1.2–3.3	0.0051
S61 Wound of wrist and hand	20 (5.3)	40 (2.2)	2.4	1.4–4.0	0.0013
T36 Poisoning/intoxication	19 (5.0)	23 (1.3)	3.9	2.2–7.1	<0.0001
O04 Medical abortion	19 (5.0)	27 (1.5)	3.3	1.9–5.9	<0.0001
F83 Mixed developmental disorder	19 (5.0)	48 (2.7)	1.9	1.1–3.2	0.0117
G43 Migraine	15 (4.0)	37 (2.1)	1.9	1.1–3.5	0.0301
H50 Strabismus	14 (3.7)	32 (1.8)	2.1	1.1–3.8	0.0209
PAE= Prenatal Alcohol Exposure, FASD= Fetal Alcohol Spectrum Disorders, RR= Risk Ratio, C.I.=Confidence Interval					

S1 Table 1.4. Congenital anomalies, 25 most common, in PAE and controls including all diagnoses in ICD-10 chapter XVII Congenital malformations, deformations (Q00-Q89) except chromosomal abnormalities (Q90-Q99)

FASD group 25 most common Q00-Q99 diagnoses	FASD N	FASD proportion in percents	PAE without FASD group 25 most common Q00-Q99 diagnoses	PAE N	PAE proportion in percents	CONTROL group 25 most common Q00-Q99 diagnoses	CONTROL group N	CONTROL proportion in percents
Q86.0	42	87,5	Q21.0	7	1,8	Q38.1	31	1,7
Q75.0	24	50	Q65.2	6	1,6	Q21.0	23	1,3
Q18.2	16	33,3	Q53.1	4	1,1	Q65.2	18	1
Q38.4	12	25	Q40.0	4	1,1	Q53.1	17	0,9
Q10.5	9	18,8	Q38.1	4	1,1	Q82.5	16	0,9
Q30.1	8	16,7	Q65.0	3	0,8	Q25.0	14	0,8
Q11.3	6	12,5	Q60.3	3	0,8	Q21.1	13	0,7
Q21.1	5	10,4	Q35.7	3	0,8	Q10.5	12	0,7
Q21.0	4	8,3	Q21.1	3	0,8	Q82.4	11	0,6
Q02	4	8,3	Q82.5	2	0,5	Q65.0	9	0,5
Q17.1	3	6,2	Q76.2	2	0,5	Q87.4	8	0,4
Q14.0	3	6,2	Q71.0	2	0,5	Q62.0	8	0,4
Q07.8	3	6,2	Q67.6	2	0,5	Q17.5	8	0,4
Q82.5	2	4,2	Q66.6	2	0,5	Q75.0	7	0,4
Q82.4	2	4,2	Q35.1	2	0,5	Q55.2	7	0,4
Q75.9	2	4,2	Q31.4	2	0,5	Q66.5	6	0,3
Q74.8	2	4,2	Q25.0	2	0,5	Q62.7	6	0,3
Q74.0	2	4,2	Q05.9	2	0,5	Q24.2	6	0,3
Q66.6	2	4,2	Q05.2	2	0,5	Q74.0	5	0,3
Q65.2	2	4,2	Q89.7	1	0,3	Q65.4	5	0,3
Q65.1	2	4,2	Q85.1	1	0,3	Q35.1	5	0,3
Q25.5	2	4,2	Q82.9	1	0,3	Q23.1	5	0,3
Q25.0	2	4,2	Q82.2	1	0,3	Q69.1	4	0,2
Q14.2	2	4,2	Q80.2	1	0,3	Q67.6	4	0,2
Q87.8	1	2,1	Q80.9	1	0,3	Q66.1	4	0,2

S1 Table 1.5. Birth weight, birth length and gestational age at birth group comparison. Statistically significant p-values are marked with a star.

	PAE with FASD (F)	PAE without FASD (P)	Controls (C)	Comparison p-values
Birth weight in grams, mean (SD)	2525.6 (630.6)	3191.1 (607.3)	3447.2 (634.1)	F-C < 0.0001 *<br>P-C < 0.0001 *<br>F-P < 0.0001 *
Birth length in cm, Mean (SD)	45.1 (3.4)	48.5 (3.2)	49.7 (2.9)	F-C < 0.0001 *<br>P-C < 0.0001 *<br>F-P < 0.0001 *

Gestational age in days at birth,<br>Mean (SD) and in pregnancy weeks + days	267.8<br>(16.8)<br>38+2	275.3<br>(17.5)<br>39+2	275.7 (16.6)<br>39+3	F-C < 0.0001 *<br>P-C = 0.3541<br>F-P = 0.0004 *
