# Supplementary material for: A register-based study of long-term health and social care costs among children with prenatal alcohol exposure
Source: PLoS One. 2026 Mar 11;21(3):e0332113. doi: 10.1371/journal.pone.0332113 (PMC12978448; doi:10.1371/journal.pone.0332113)
Supplement: S2 Appendix — Out-of-home care types and costs. (PDF) [file pone.0332113.s002.pdf]

S2. Out-of-home care

2.1 Out-of-home care placement types

01 Foster care in a Relative/Kinship placement

02 Foster care in a foster family

03 Professional group home with a group home permit

04 Professional group home with an institutional permit

05 Children's home

06 Family rehabilitation unit

07 Reform school

08 Institutional/residential care for persons with substance use disorder

09 Institutional/residential care for persons with intellectual disability

10 Supported living with parent under surveillance

11 Supported independent living

12 Other type of out-of-home care

2.2 Prices for out-of-home care

S2 Table 2.1. Out-of-home care cost per day for different care types (https://www.kuusikkokunnat.fi/lastensuojelu_raportit)

Placement type	Cost/day 2005 €	2006	2007	2008	2009	2010	2011	2012	2013	2014	2015	2016
Foster family, (1, 2,10-12)	44	45	46	56	62	64	75	81	83	88	93	102
Professional group home (3-4)	144	133	155.7	165.4	175	159	182	169	147	185	182	191
Institution (5-9)	206.8	223.7	225.5	235.8	244.5	252.5	269	272.5	293.5	317.5	333	309

Price for institutional care was calculated as an average of municipal and out-sourced institutional care.

For years 2005-2008, the price for institutional care was calculated as averages for the three main cities of the residence in cases Helsinki, Espoo and Vantaa, because the average for main six cities was not available. The price for professional group homes was missing for 2008, it was estimated using by averaging years 2007 and 2009.

As prices before 2005 were not available, they were estimated using social and health care price index. Finally, all prices were then index corrected to 2023 price for comparison.
