# Supplementary material for: A register-based study of long-term health and social care costs among children with prenatal alcohol exposure
Source: PLoS One. 2026 Mar 11;21(3):e0332113. doi: 10.1371/journal.pone.0332113 (PMC12978448; doi:10.1371/journal.pone.0332113)
Supplement: Attachment — Submitted filename: Response_to_reviewers.docx [file pone.0332113.s004.docx]

Response to Reviewers

Editor: Thank you for submitting your manuscript to PLOS ONE. After careful consideration, we feel that it has merit but does not fully meet PLOS ONE’s publication criteria as it currently stands. Therefore, we invite you to submit a revised version of the manuscript that addresses the points raised during the review process.

Our response: We thank you for the positive feedback. Below we give our response to the specific editorial comments and the comments from the reviewers.

1. Please ensure that your manuscript meets PLOS ONE's style requirements, including those for file naming.

Our response: Files have been named according to the instructions.

 2. We note that you have indicated that there are restrictions to data sharing for this study. For studies involving human research participant data or other sensitive data, we encourage authors to share de-identified or anonymized data. However, when data cannot be publicly shared for ethical reasons, we allow authors to make their data sets available upon request. For information on unacceptable data access restrictions, please see [http://journals.plos.org/plosone/s/data-availability#loc-unacceptable-data-access-restrictions](http://journals.plos.org/plosone/s/data-availability#loc-unacceptable-data-access-restrictions).

Before we proceed with your manuscript, please address the following prompts:

a) If there are ethical or legal restrictions on sharing a de-identified data set, please explain them in detail (e.g., data contain potentially identifying or sensitive patient information, data are owned by a third-party organization, etc.) and who has imposed them (e.g., a Research Ethics Committee or Institutional Review Board, etc.). Please also provide contact information for a data access committee, ethics committee, or other institutional body to which data requests may be sent.

Please update your Data Availability statement in the submission form accordingly.

 Our response: Data availability clause has now been refined: “The data that support the findings of this study are available from the Hospital District of Helsinki and Uusimaa (HUS). However, restrictions apply to the availability of these sensitive data, which were used under license for the current study. Only those researchers who are named in the study permissions have access to the data, and no part of the data can be shared or placed in public repositories. Similar data can be applied from Findata Finnish Social and Health Data Permit Authority Findata https://findata.fi/en/. Link to the current legislation: https://findata.fi/en/services-and-instructions/legislation/.”

3. Please include captions for your Supporting Information files at the end of your manuscript, and update any in-text citations to match accordingly. Please see our Supporting Information guidelines for more information: <http://journals.plos.org/plosone/s/supporting-information>.

Our response: Captions for Supporting Information have been added.

 4. Please remove all personal information, ensure that the data shared are in accordance with participant consent, and re-upload a fully anonymized data set.

Our response: Due to the strict research permit, no information that relates to individuals has been uploaded.

5. If the reviewer comments include a recommendation to cite specific previously published works, please review and evaluate these publications to determine whether they are relevant and should be cited. There is no requirement to cite these works unless the editor has indicated otherwise.

Our response: There were no recommendations for previously published works.

6. Please review your reference list to ensure that it is complete and correct. If you have cited papers that have been retracted, please include the rationale for doing so in the manuscript text, or remove these references and replace them with relevant current references. Any changes to the reference list should be mentioned in the rebuttal letter that accompanies your revised manuscript. If you need to cite a retracted article, indicate the article’s retracted status in the References list and also include a citation and full reference for the retraction notice.

Our response: The reference list has been reviewed.

Reviewer #1: while the methodology of the study suggests a retrospective/non-concurrent design with the children being identified based on exposure and followed up for outcome the analysis is of a case control design. The measure of association we would look for would be relative risk rather than odds ratio. However, the data is interesting and requires a resubmission after considering the suggestions.

Our response: We thank the reviewer for the interest in our study. We have now replaced odds ratios with risk ratios in group comparisons.

Reviewer #2: This is a considered and well-written paper demonstrating a high level of expertise on a complex and nuanced subject. The methodology is sound, and the findings are interesting with potential for international impact. There are some minor changes I would recommend before publication:

Our response: We thank the reviewer for the interest in our study and for several useful suggestions to improve our manuscript. We give our point-by-point responses to the specific suggestion below.

Terminology Clarification
The term “Out-of-home care” may be problematic from an international perspective, as it could be misinterpreted to mean hospital admission. It appears the authors are referring to children under the care of the state or local authority, including foster care and possibly specialized mother-and-baby units? Clarifying this terminology early on is important to accessibility to a global audience.

Our response: Out-of-home care has now been clarified (in abstract line 30) to include placements in foster or residential care. It has also been clarified similarly in Introduction (line 76) and in Discussion (line 357).

International Comparison to Sweden
The inclusion of figures from Sweden in the introduction. It would strengthen the paper to briefly explain why Sweden is a relevant comparator and highlighting key similarities and differences between the two countries in terms of policy, demographics, or care systems for example.

Our response: This is an excellent suggestion, as the economic, societal, historic, demographic, cultural and geographical similarities between Sweden and Finland might not be self-evident to international readers. Brief explanation of similarities and comparability between Sweden and Finland has now been added to Introduction (lines 63-64). The information about Sweden having a comparable social and healthcare system to Finland has also been added to Discussion (line 398).

Table 1 – Combined Risk Factors
It is good that Table 1 includes data on pre-pregnancy alcohol use and unplanned pregnancies. If possible, consider adding a combined metric showing cases where both factors were present. This could offer valuable insight into alcohol consumption prior to the recognition of pregnancy. This is an important factor in future prevention of PAE health promotion efforts.

Our response: The combination of unplanned pregnancy and daily or weekly alcohol use have now been added as additional rows in Table 1.

PAE and Control Group Considerations
The paper appropriately acknowledges that the control group may include individuals with prenatal alcohol exposure (PAE), given the limitations of self-reporting. This along with the recognition that some individuals in the PAE group may have undiagnosed FASD is important. It would be good to see the limitations of recording PAE in general but also specific to this collection method in more detail in the discussion.

Our response: Unrecognition is a very important topic. More discussion about presence of probable undiagnosed FASD in PAE population has been added (lines 452-456). Also, description of problems in recognition and recording of PAE in antenatal care and limitations of register data has been added to limitations (lines 463-472).

Subgroup Analysis – FASD-like Characteristics
The discussion refers to a subgroup within the PAE group that exhibits characteristics similar to FASD. It would be beneficial to describe this subgroup in greater detail and, if feasible, include this data in table form to support.

Our response: This subgroup of PAE exhibiting typical symptomatic diagnoses and cost patterns has now been addressed in the Results section as text (lines 327-331) and the new Figure 6. Its similarity to FASD diagnosed both in cost and diagnose patterns had been added to Discussion (lines 393-396)

Exposure Patterns and Outcomes
I recommend expanding the discussion to explore whether differences in exposure patterns—such as episodic drinking during the first trimester—might influence outcomes like depression and anxiety. Some evidence suggests that timing and pattern of exposure may be as important as dose.

Our response: This is a very interesting and important topic warranting more research. The Discussion has been expanded to include this subject (lines 382-386).

Cost Comparisons
In the discussion (and the introduction) including a comparison of the total costs associated with FASD and PAE to those of other prevalent conditions would provide useful context and underscore the broader public health implications.

Our response: This is another great suggestion for improvement. Comparison with costs of childhood asthma and epilepsy have been added (lines 66-68 and 401-404). Unfortunately, cost analyses for many chronic childhood conditions are still lacking.

Reduced Injury Rates in FASD Group
The finding of reduced injury rates in the FASD group is intriguing. Are there any known factors specific to Finland that might explain this? A comparison with data from other countries and further exploration in the discussion would be valuable. This could be an area for further research which could be highlighted in the paper too.

Our response: This is indeed an intriguing and rather surprising finding that requires further research. Earlier studies have indicated that early diagnosis of FASD has protective effect on many adverse health outcomes, but we could not find other studies researching or showing protective effect on traumatic injuries. Discussion about some possible explanation this association in Finland is added (lines 361-372).
