# Supplementary material for: A register-based study of long-term health and social care costs among children with prenatal alcohol exposure
Source: PLoS One. 2026 Mar 11;21(3):e0332113. doi: 10.1371/journal.pone.0332113 (PMC12978448; doi:10.1371/journal.pone.0332113)
Supplement: Attachment — Submitted filename: Response_to_reviewers_auresp_2.docx [file pone.0332113.s005.docx]

Response to Editor and Reviewer

**Editor**:

Thank you for submitting your manuscript to PLOS ONE. After careful consideration, we feel that it has merit but does not fully meet PLOS ONE’s publication criteria as it currently stands. Therefore, we invite you to submit a revised version of the manuscript that addresses the points raised during the review process.

1) Reviewer 1 has asked that all references to case control studies be removed, since the study is a non-concurrent cohort study.

Our response: We thank the editor for the encouragement. Following the reviewers suggestion, we have removed all instances where we refer our study as “case-control”. Additionally, below we give our response to the comments from the reviewers.

**Reviewer #1**:

1) PLEASE REMOVE ALL REFERENCES TO CASE CONTROL STUDIES AS THIS IS A NON-CONCURRENT COHORT STUDY. OTHERWISE tHIS MANUSCRIPT IS FINE

Our response: We thank the reviewer for the continuing interest in our manuscript and for the time spent reviewing it. We have removed all instances of “case-control” from the title and the text, as requested.

**Reviewer #2**:

1) Overall this is an very interesting piece of research with impactful findings. I can see my comments have been addressed satisfactorily and I am happy to recommend publishing this paper with a few minor amendments.

Our response: We thank the reviewer for the positive response and for the time spent reviewing the manuscript.

2) Please consider rephrasing the line 42 of abstract. "..support and rehabilitation for birthmothers with alcohol consumption during and after pregnancy" (and line 475 of conclusion) to be less blaming and less directed at birth mothers, also the term rehabilitation used in this line heavily suggests all prenatal alcohol exposure results from dependent drinking which would be incorrect.
It may be helpful to phrase this as "prevention of alcohol exposed pregnancies and increased support need" for example.

Our response: We have rephrased the passages in abstract and conclusions following the suggestion.

3) Line 119 where it reads "mental and behavioural", I think should be "mental health and behavioural".

Our response: The phrase “mental and behavioural disorders” refers to the name of a class of diagnoses in ICD-10 classification.
